# Evaluation of Antibacterial Activity of a Bioactive Restorative Material Versus a Glass-Ionomer Cement on Streptococcus Mutans: In-Vitro Study

**DOI:** 10.3390/dj11060149

**Published:** 2023-06-08

**Authors:** Giulio Conti, Federica Veneri, Francesca Amadori, Alba Garzoni, Alessandra Majorana, Elena Bardellini

**Affiliations:** 1 Department of Medicine and Surgery, School of Dentistry, University of Insubria, Via Ravasi 2, 21100 Varese, Italy; 2 Department of Surgery, Medicine, Dentistry and Morphological Sciences with Transplant Surgery, Oncology and Regenerative Medicine Relevance, Unit of Dentistry & Oral-Maxillo-Facial Surgery, University of Modena and Reggio Emilia, Via del Pozzo, 41124 Modena, Italy; 3 Department of Medical and Surgical Sciences and Public Health, School of Pediatric Dentistry, University of Brescia, Pl. Spedali Civili 1, 25123 Brescia, Italy

**Keywords:** bioactivity, restorative material, glass ionomer cement, antimicrobial activity, secondary caries

## Abstract

Background: Dental caries management consists of both preventive and restorative approaches. Pediatric dentists can rely on many techniques and materials to restore decayed teeth, but a high failure rate is still observed, mainly due to secondary caries. New restorative bioactive materials combine the mechanical and aesthetic characteristics of resinous materials with the capability to remineralize and the antimicrobial properties of glass ionomers, thus counteracting the occurrence of secondary caries. The aim of this study was to assess the antimicrobial activity against *Streptococcus mutans* of a bioactive restorative material (ACTIVA™ BioActive-Restorative™-Pulpdent©) and a glass ionomer cement with silver particles added (Ketac™ Silver—3M©), using agar diffusion assay. Methods: Each material was formed into disks of 4 mm in diameter, and four discs of each material were placed on nine agar plates. The analysis was repeated seven times. Results: Both materials showed statistically significant growth inhibition properties against *S. mutans* (*p* < 0.05). The difference in the effectiveness of the two materials was not statistically significant. Conclusion: Both ACTIVA™ and Ketac™ Silver can be recommended since both are similarly effective against *S. mutans*. However ACTIVA™, given its bioactivity and better aesthetics and mechanical properties compared to GICs, may provide better clinical performance.

## 1. Introduction

Dental decay is a multifactorial oral disease that affects approximately 621 million children globally, and it is estimated to be the 10th most common disease in children; thus, it is considered a public health issue [1]. 

Untreated dental caries can gradually worsen and lead to pain, infection, swelling, and other debilitating symptoms. These symptoms not only have a negative impact on oral health, but they also reduce the quality of life of both children and their families. Oral pain, especially in children, can disrupt sleep patterns, eating habits, physical development, and educational progress [2,3,4,5]. In fact, dental conditions are responsible for more than two million missed school days per year in the United States [4]. Parents are five times more likely to seek urgent medical attention for their children due to dental pain compared to other health issues [2,4].

The management of dental caries involves both preventive and restorative approaches. Primary preventive strategies include adopting appropriate dietary habits and implementing effective oral hygiene procedures at home [6], However, considering the current prevalence of dental caries among children, these approaches are not sufficiently effective, as they can be demanding for many children and parents to incorporate into their daily routine practices [6]. Therefore, in addition to home-care methods, professional preventive approaches include topical fluoride applications, such as gels and varnishes; the use of other remineralizing agents, such as nano-hydroxyapatite and casein; calcium phosphate compounds; and the application of modern pit and fissure sealants [7,8,9,10,11,12]. 

For the treatment of active cavitated lesions, restoration with suitable materials is necessary after the removal of decayed and demineralized tissue [13,14]. 

Pediatric dentists can rely on many techniques and materials to restore decayed teeth: composite resins (CRs), glass-ionomer cements (GICs), resin-modified glass-ionomer cements (RMGICs), compomers or polyacidic-modified composite resins (PAMCRs), and preformed crowns. While composite resins offer improved aesthetic and mechanical properties compared to older materials such as amalgam, there are concerns regarding their longevity and their reliability in suboptimal clinical situations, especially in pediatric restorative dentistry [13]. Long-term analyses have reported a large number of failures, primarily due to the development of secondary caries. The longevity of restorations depends on several factors, such as the operator’s skill and patient characteristics [15,16,17].

The main risk factors leading to secondary caries are mechanical seal failure and margin gaps, which allow for biofilm adsorption, progressive wear of the material, bacterial infiltration, and subsequent demineralization [17]. Obtaining a tight seal through a reliable adhesive interface at the restoration margins can mitigate such issues; however, clinical management of pediatric patients often complicates the achievement of proper isolation of the operating field, thus hampering the effectiveness of traditional adhesive systems [16,17].

Therefore, desirable properties for pediatric restorative materials indeed include the ability to provide a reliable adhesive interface, to exert antimicrobial activity against cariogenic bacteria, such as *Streptococcus mutans,* and to promote tooth remineralization to delay and possibly prevent the occurrence of secondary caries [14,16]. 

Glass ionomer-based materials have shown good secondary caries-inhibiting properties thanks to their antibacterial activity, due to the low pH before setting and their ability to release fluoride over the long term [18]. Additionally, they are able to chemically bond to the tooth structure through ion exchange, without the need for additional adhesive systems [18,19,20]. Thanks to these properties, GICs potentially reduce the risk of the onset of secondary carious lesions, and they are the material of choice, compared to conventional CRs, in a moist environment when proper isolation through the placement of a rubber dam cannot be performed or in patients who are uncooperative due to age or disability. However, they have shown poor mechanical properties, with low resistance to wear and flexure [21]. 

To improve the mechanical behavior of GIC-based materials, RMGICs were introduced, providing a good compromise of aesthetics, functional, and mechanical performances, but on the other hand, compared to traditional GICs, RMGICs were found to be less biocompatible and more cytotoxic to the cells of the pulp due to the presence and diffusion through the dentin of a specific monomer component [19,22].

More recently, there has been a significant focus on the development of a new generation of biocompatible restorative materials that have therapeutic functions. These materials have the remarkable capability to induce remineralization of dental residual tissue surrounding cavities, while also exhibiting strong antimicrobial properties. These innovative materials are referred to as bioactive, setting them apart from conventional bioinert composite resins [23,24,25]. In the broadest sense, bioactivity refers to a defining characteristic of materials that possess the ability to exert a biological effect or demonstrate biological activity by forming a bond with vital tissues [26]. In the context of tissue engineering, the term *bioactive* extends to encompass the cellular effects triggered by the release of biologically active substances and ions from the biomaterial itself. A prime example of this phenomenon is observed with glass ionomer materials [27].

When a material is considered bioactive, it implies that it can interact with biological systems in a manner that elicits specific responses. These responses may involve the stimulation of cellular processes, such as cell proliferation, differentiation, or the production of extracellular matrix components. Furthermore, bioactive materials may exhibit bioactivity through the release of growth factors, cytokines, or other biologically active molecules that influence cellular behavior and tissue regeneration processes. In the realm of tissue engineering, bioactive materials hold significant promise since they can actively participate in the healing and regeneration of damaged or diseased tissues. By leveraging the release of biologically active substances and ions, these materials can promote cell adhesion, migration, and proliferation, facilitating tissue integration and fostering favorable host–material interactions. This unique property makes bioactive materials a valuable asset in the development of advanced biomaterials for various biomedical applications. 

Among the materials that demonstrate bioactivity, glass ionomer materials exemplify this concept vividly. Due to their composition and specific chemical properties, these materials have the capacity to release ions, such as fluoride, calcium, and phosphate, which have positive impacts on the surrounding biological environment. This ion release contributes to several beneficial effects, including remineralization of tooth structure, inhibition of bacterial growth, and prevention of secondary caries formation. Such bioactivity makes glass ionomer materials particularly suitable for applications in dentistry and orthodontics. Bioactivity represents a crucial attribute of materials that can exert biological effects, establish bonds with vital tissues, and trigger cellular responses. In tissue engineering, bioactive materials play a pivotal role in promoting tissue regeneration and repair through the release of biologically active substances and ions. Glass ionomer materials serve as an excellent example of bioactive materials given their ability to induce cellular effects via the release of biologically active components. In medicine, bioactivity encompasses all interactions of materials with living cells and tissues, including pharmaceutical effects [26]. In biomaterial science, bioactivity denotes the ability of the material to form hydroxyapatite on the surface of the material, in vivo and in vitro [26]. This ability is a qualitative property of the material, resulting from its chemical composition and surface structure, and the qualities of the surrounding microenvironment, which allow for the mineralization process to occur [27].

Factors contributing to the failure of the restoration are related to the greater accumulation of biofilm on the surface of the resin compared to other restorative materials (e.g., GIC and amalgam) and the inherent weakness of the tooth-restorative interface that may lead to the formation of microgaps and microleakage, causing proneness to secondary caries formation [28]. The bioactivity of dental materials, thus, refers to their potential to create a specific biomineralization-inducing bond with the tooth substrate and to counteract cariogenic bacteria. Bioactivity has a major impact, especially in pediatric dentistry, as it allows for reliable conservative approaches on deep carious lesions, such as selective caries removal, and it can effectively prevent the occurrence of secondary caries [25]. Additionally, an important feature of these recent materials is their high chemical and physical biocompatibility since they contain no bisphenol A, no bis-GMA, and no BPA derivatives, and they mimic the natural tooth tissues, showing an elasticity modulus similar to that of dentin and shock-absorbing mechanical characteristics [25,26,27].

ACTIVA™ BioActive-Restorative™ (Pulpdent^®^ Corporation, Watertown, MA, USA) is a recent bioactive material that combines the mechanical and aesthetic characteristics of resinous materials with the caries-inhibiting and antibacterial properties of GICs [25,29,30,31].

The purpose of this in vitro study was to evaluate and to compare ACTIVA™ BioActive-Restorative™ (Pulpdent^®^) and Ketac™ Silver (3M™, St. Paul, MN, USA) for their antibacterial properties against *Streptococcus mutans.* A semi-quantitative evaluation of the growth inhibition activity, by agar diffusion assay, was chosen to test the null hypothesis (H_0_) that there are no differences in the inhibition halo values between the two tested materials.

## 2. Materials and Methods

ACTIVA™ BioActive-Restorative™(Pulpdent)© was tested as a bioactive restorative material and Ketac™ Silver (3M™) as a silver-reinforced GIC.

*Streptococcus mutans* strain 25175 (MS 25175, Microbiologics, North St. Cloud, MN, USA) was tested as the main cariogenic agent in this in vitro study. 

All procedures were performed in a sterile environment. Lyophilized bacterium (1 mL) was placed in culture medium (Tryptic Soy Broth—TSB) for stabilization. 

MS 25175-TSB suspension was incubated at 37 °C for 48 h, according to standard inclusion techniques to obtain a solution with density of 3 × 10^8^ cells/mL (=1 McFarland). A known amount of solution (100 μL) was placed in a 10-mL tube with 0.3% soft agar. The tube content was then transferred to a Petri plate containing blood agar (code 43041-bioMérieux Italia Spa, Bagno a Ripoli (FI) 50012, Italy). The plate was allowed to settle for 4 min. The disks of material were prepared under a laminar flow hood with sterile instruments on a glass to obtain 4 mm-diameter disks.

The ACTIVA™ disks were extruded using the specific ACTIVA™ tip and light-cured for 30 s. Ketac Silver™ was properly mixed for 8 s and extruded using the specific dispenser to obtain disks that were allowed to harden for 4 min. The build-up of the tested materials was measured at a room temperature of 25 ± 1 °C.

Ultimately, four disks of the same material were placed on each plate, for a total of nine plates per material.

Plates were incubated at 37 °C for 48 h. After incubation, the inhibition halo was measured for each disk of material. The halo of inhibition was measured at its largest diameter, using calipers under 4.5× magnification. The experimental protocol was performed in seven repetitions.

All data were recorded in Microsoft Excel datasheets. Statistical analysis was performed using IBM SPSS Statistics software (v25, Inc., Chicago, IL, USA). Minimum, maximum, and mean values were calculated and considered for the analysis. After verifying the normal distribution of data using the Kolmogorov–Smirnov test, Student’s parametric *t*-test was applied to compare the variables. The significance level was set at *p* < 0.05.

## 3. Results

For each tested material, ACTIVA™ BioActive-Restorative™ (Pulpdent^®^) and Ketac™ Silver (3M™), a total of 252 inhibition halos were measured (four disks per plate, nine plates, seven repetitions) (Supplementary Table S1).

Given the microbiological variability in the measurements of inhibition halos found during the first experimental tests (Supplementary Table S1), seven repetitions were performed to collect a large number of data to be analyzed and to reach definitive and consistent conclusions. 

ACTIVA™ BioActive-Restorative™ showed zones of growth inhibition with a minimum value of 6 mm and a maximum of 12.5 mm; the mean of the halo was 8.1 mm with a standard deviation of 1 mm.

KETAC™ Silver (3M)© showed zones of growth inhibition with a minimum value of 6 mm and a maximum of 13 mm; the mean was 8.5 mm with a standard deviation of 1.2 mm.

**Figure 1:**
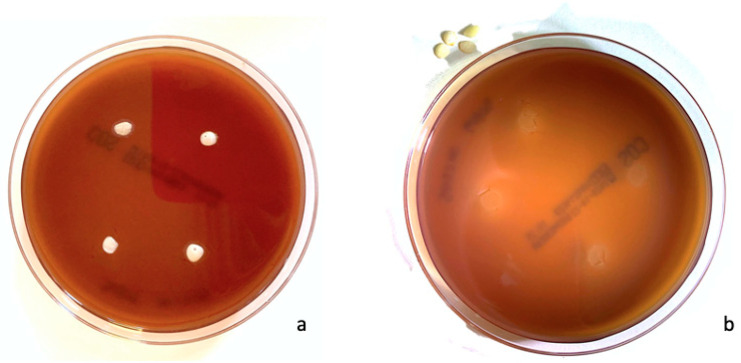
(**a**) Plate with ACTIVA™ BioActive-Restorative™ disks after incubation at 37 °C for 48 h. (**b**) The same plate after removal of the disks of ACTIVA™ BioActive-Restorative™. Growth inhibition halos are visible.

**Figure 2:**
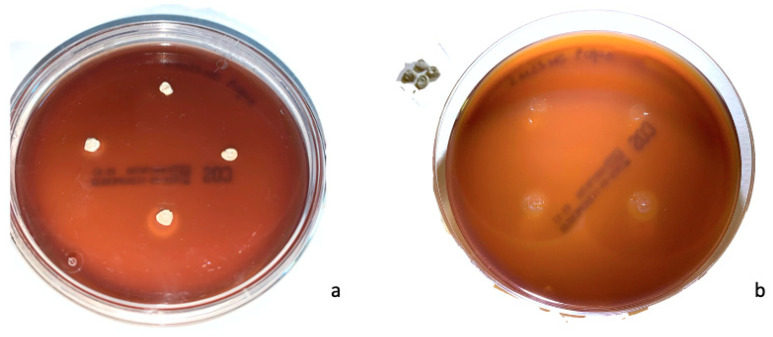
(**a**) Plate with Ketac™ Silver disks after incubation at 37 °C for 48 h. (**b**) The same plate after removal of the disks of Ketac™ Silver. Growth inhibition halos are visible.

Sample images of growth inhibition halos of ACTIVA™ BioActive-Restorative™ and Ketac™ Silver are shown in Figure 1 and Figure 2, respectively. The results are summarized in Table 1. 

Both materials showed statistically significant activity of growth inhibition against *S. mutans.* However, the difference in the effectiveness of the two materials was not statistically significant (*p* = 0.83); therefore, the null hypothesis (H_0_) was not rejected.

## 4. Discussion

The present study was conducted to test the antibacterial activity of two different restorative materials used in pediatric dentistry, both to treat cavitated lesions and to prevent secondary caries, by measuring the halos of growth inhibition of *Streptococcus mutans* on Petri plates through agar diffusion assay.

Poor mechanical properties, including low bond strength, and interfacial microleakage, can account for restoration failures over time and secondary caries occurrence [32]. However, GICs have shown reliable interfacial properties, with durable bond strength and low microleakage [32].

Biofilm formation at the restoration interface is nevertheless a major cause of secondary caries [33,34]. 

Biofilm progressively accumulates at the restoration interface, resulting in significant surface deterioration and roughening of the material, ultimately leading to microleakage and restoration failure due to secondary caries [35]. In light of this fact, this study aimed at testing the antibacterial activity of two pediatric restorative materials.

Many restorative materials with different characteristics and properties are available on the market, some of which are specifically recommended for pediatric dentistry, such as GICs and bioactive composites [17]. Among these materials, those showing antibacterial activity and remineralizing properties are the most suitable [36].

Ketac™ Silver was chosen over a conventional GIC as the most appropriate material for the purpose of this study thanks to its improved antibacterial activity against cariogenic biofilm, provided by the addition of silver [33,37,38]

Silver ions exert effective antimicrobial activity through three major mechanisms: inhibition of cellular respiration and disruption of metabolic pathways by generating reactive oxygen species, disruption of DNA replication, and direct damage of the bacterial cell wall through reactions with peptidoglycan [39]. Additionally, the addition of silver particles to GICs does not negatively affect their mechanical and physical properties [40,41]

As a side note, according to the manufacturer’s instructions, the setting time for Ketac™ Silver is generally 5 min from the beginning of mixing at room temperature within 23 °C, while it is accelerated at higher room temperatures. Since the curing and setting of the tested materials occurred at a room temperature of 25 ± 1 °C, we chose a setting time of 4 min.

Although multispecies biofilms, composed by both bacteria and fungi, such as *Candida albicans,* are now known to play an important role in caries pathogenetic process, the main cariogenic agent is *Streptococcus mutans,* and it was thus chosen for this study [42,43]. *S. mutans* produces acids through the metabolization of dietary sugars, thus lowering the pH of the oral cavity to less than the solubility limit of dental hard tissue [42,44].

Fluoride release from restorative dental materials exerts a cariostatic effect since it can prevent caries development thanks to its ability to inhibit the growth of *S. mutans,* thus hindering the production of acids [45]. Additionally, it promotes dental hard tissue remineralization through the formation of fluorapatite, probably thanks to its improved crystal stability and more compact structure, as demonstrated by the lower solubility product (Ksp) of fluorapatite compared to hydroxyapatite [46]. 

In light of this fact, GICs are considered the gold standard among fluoride-releasing restorative materials, although they are not the only ones on the market with this feature: the amount of fluoride absorbed by enamel adjacent to GIC fillings is twice as much as that absorbed in enamel adjacent to fluoride-containing composite resins [47,48].

Additionally, with regard to antibacterial activity, GICs are considered the best choice due to the low pH of the cement prior to curing and their ability to release fluoride [18,19,20].

Several in vitro studies have been conducted to test the growth inhibition capacity against *S. mutans* of different commercially available GICs, and although the halo of growth inhibition has different measures depending on the specific brand formulation, it is always present [49,50].

A systematic review analyzing the literature to find specific indications regarding which material is the most suitable for the treatment of carious lesions on deciduous teeth highlighted a higher risk of failure for conventional GICs compared to RMGICs; however, a lack of strong evidence prevented the authors from recommending the use of one material rather than another [51]. GICs’ mechanical and aesthetic properties are indeed inferior to those of composite resins, and although RMGICs improve on the aesthetic and mechanical qualities of GICs while maintaining the advantages of self-adhesion and fluoride buffering, lower biocompatibility compared to conventional GICs has been reported [18,36].

To overcome these issues, research has recently focused on the development of a new generation of bioactive restorative materials that have additives able to remineralize the remaining dental tissue surrounding the restoration and that offer improved antimicrobial characteristics, thus enhancing their preventive action against secondary caries [52]. 

The addition of calcium phosphate (CaP) particles to composite resins has shown promising results, with these resins able to effectively remineralize dental hard tissues in vitro [53]. CaP particles range between 1 μm and 55 μm in size and are able to release large numbers of calcium (Ca) and phosphate (P) ions [54]. However, CaP-composites showed poor mechanical properties, with a flexural strength remarkably lower than that of traditional resin composites, thus making them unacceptable for everyday clinical practice [55]. 

More recently, CaP particles of smaller size (100 nm) have been synthetized that exhibit the same ion release profile, along with significantly better mechanical properties [56]. Composite-containing amorphous calcium phosphate nanoparticles (NACP) showed a good degree of remineralization in vitro, achieving a degree of remineralization four times greater than that of a commercially available fluoride-releasing composite [57].

NACP composites and adhesives have shown the ability to raise the pH from 4 to more than 6 quickly, while GICs are not able to raise it to greater than 4 [58]. Despite these promising results, a major limitation of CaP- and NACP-containing resins was that ion release lasted only about two months after application of the resin [59]. To overcome this problem, composites and adhesives have recently been tested that are capable of recharging with CaP through local weekly application, thus acquiring the ability to potentially inhibit caries formation in the long term [59].

Regarding antibacterial activity, the addition of compounds, such as quaternary ammonium, has provided resin materials with antibacterial properties, which were until then exclusive characteristics of GICs [60]. Adhesive materials with quaternary ammonium dimethacrylate have also been formulated that are able to reduce residual bacterial load in the prepared cavity and inhibit bacterial growth at the tooth–restoration interface [61]. 

The adhesion of bacteria to the dental restoration is achieved by the formation on the tooth of a film of proteins absorbed by saliva. Therefore, the development of materials capable of blocking the formation of this protein layer would be desirable [16].

Hydrophilic surfaces are more resistant to protein and bacterial adhesion than hydrophobic ones [62]. For this purpose, the use of a specific type of methacrylate was suggested (methacrylate-oxyethyl-phosphorylcholine—MPC) since it is a common biocompatible and hydrophilic polymer for biomedical use [63].

MPC shows excellent resistance to protein and bacterial adhesion, and tested composites with 3% MPC added have achieved excellent results in counteracting bacterial adhesion without affecting their mechanical properties [64].

By implementing hydrophilic features with antibacterial and remineralizing agents, as established by the aforementioned research, modern bioactive materials are able to combine the mechanical and aesthetic characteristics of resinous materials with the optimal remineralizing and antibacterial activities of GICs. ACTIVA™ BioActive-Restorative™ (Pulpdent)© is a resin-based material defined as a bioactive composite with a glass ionomer component and modified ion-releasing resin base, as it contains diurethane, other methacrylates, polyacidic acids, amorphous silica, sodium fluoride [65]. 

Biocompatibility studies have shown that ACTIVA™ is able to promote cell adhesion and migration of odontoblasts and fibroblasts, increasing their metabolic activity [66]. 

Additionally, according to the manufacturer, this material establishes a dynamic interchange system of phosphate, calcium, and fluoride ions with saliva and the residual tooth structure: this process allows for a bond between the material and the tooth, providing it with hydrophilicity properties. 

An in vitro study by Porenczuk et al. compared the fluoride-releasing capacity of ACTIVA™ with a RMGIC (Ketac Molar Quick Aplicap™, 3M™, St. Paul, MN, USA) and a nanohybrid composite resin (Tetric EvoCeram™, Ivoclar Vivadent, Schaan, Liechtenstein). The fluoride release was higher for the RMGIC (20,698–54,118 ppm), intermediate for ACTIVA™ (1236–15,552 ppm), and minimal for the composite resin (0.370–1.148 ppm), as expected [47]. 

An in vitro study [67]. analyzed the antimicrobial activity and pH of ACTIVA™ compared with tricalcium silicate cements, namely Mineral Trioxide Aggregate high plasticity™ (Angelus, Lindóia Londrina, PR, Brazil)© and iRoot BP Plus™ (BioCeramix Inc., Vancouver, BC, Canada)©, against the cocci *E. faecalis*, *E. faecum, S. aureus,* and *S. mutans* and the fungus *C. albicans*. Tricalcium silicate cements have shown a significantly greater inhibitory effect than ACTIVA™ toward cocci, while the latter had no inhibitory activity toward *C. albicans*. The pH, measured at 5 and 60 min after the formation of the suspension formation, was markedly basic for tricalcium silicate cements, while it remained close to neutral for ACTIVA™. However, these results are consistent with the two tricalcium silicate cements being specifically designed for endodontic, rather than restorative, use [67].

Many studies have investigated the mechanical, aesthetic, bioactive, and ion releasing properties of ACTIVA™, finding promising results [68,69,70,71]. Based on the results of this study, ACTIVA™ exerts an effective antibacterial activity against *S. mutans*, comparable to that of GIC Ketac™ Silver. Nevertheless, it must be pointed out that the absence of saliva inherent in this in vitro setting prevented the materials from reproducing the normal ion exchange that occurs in clinical settings, thus possibly affecting the outcomes. Additionally, further studies should assess the antibacterial activity of bioactive materials against multispecies biofilms to reproduce an experimental model more similar to the clinical environment and to gain a deeper understanding of the antibacterial properties in vivo. Similar to other studies, the agar disk diffusion method was chosen to evaluate the antibacterial effects of two materials (i.e., Ketac™ Silver and ACTIVA™ Bioactive) against *S. mutans* by measuring the diameter of the inhibition halo [72,73,74]. The semi-quantitative nature of the chosen method is a potential limitation of this study, as the results may be influenced by the specific solubility and diffusion properties of the tested materials [74]. However, since such properties are also expected to impact their clinical behavior, it is reasonable to say that these results may to some extent reflect their actual antibacterial activity. Indeed, future studies are encouraged to further quantitatively compare these materials.

## 5. Conclusions

Within the limitations of this in vitro study, it can be concluded that both ACTIVA™ BioActive-Restorative™(Pulpdent)© and glass-ionomer Ketac™ Silver (3M)© exert an effective antibacterial activity against *S. mutans*. Given the lack of significant differences in the antibacterial efficacy between the tested materials, further quantitative and clinical studies are required. 

The use of both ACTIVA™ and Ketac™ Silver can be recommended in pediatric dentistry clinical practice, especially in patients at high risk for developing secondary caries; however, considering its pronounced bioactivity and better aesthetic and mechanical properties compared to GICs, ACTIVA™ might provide higher clinical performance and hence be overall the more valid option.

## Figures and Tables

**Table 1:** Results of growth inhibition testing, shown as minimum (Min) and maximum (Max) values, means, and standard deviations (SDs).

Material	Min [mm]	Max [mm]	Mean	SD	*p* ^a^	
ACTIVA™	6.0	12.5	8.1	1.0	<0.0001 *	0.83
Ketac™ Silver	6.0	13.0	8.5	1.2	0.0002 *	

^a^ Student’s two-sided *t*-test. * statistically significant, *p* < 0.05.

## Data Availability

The data used to support this study are available from the corresponding author upon request.

## Supplementary Materials

The following supporting information can be downloaded at: https://www.mdpi.com/article/10.3390/dj11060149/s1. Table S1: Inhibition halos measures (4 disks per plate, 9 plates, 7 repetitions). For ACTIVA™ BioActive-Restorative™ (Pulpdent^®^) and for KETAC™ Silver (3M™).
