# Supplementary material for: Evaluation of Antibacterial Activity of a Bioactive Restorative Material Versus a Glass-Ionomer Cement on Streptococcus Mutans: In-Vitro Study
Source: Dent J (Basel). 2023 Jun 8;11(6):149. doi: 10.3390/dj11060149 (PMC10297256; doi:10.3390/dj11060149)
Supplement: Supplementary file 1 [file dentistry-11-00149-s001.zip › dentistry-2310325-supplementary.pdf]

Supplementary Table S1. a-b-c-d-e-f-g Inhibition halos measures (4 disks per plate, 9 plates, 7 repetitions). for, ACTIVA™ BioActive-Restorative™(Pulpdent®) and for KETAC™ Silver (3M™)

a) Test 1 – First repetition

Test 1-1

Activa	Ketac
6,5	12
7	8
9,5	8,5
10	6,5

Test 1-2

Activa	Ketac
7,5	13
7	9,5
7,5	7,5
7	8

Test 1-3

Activa	Ketac
6,5	7,5
7	7,5
8,5	6
8	9

Test 1-4

Activa	Ketac
9	10
9	7
8	8,5
7,5	11

Test 1-5

Activa	Ketac
7	8
6,5	8,5
8	11
6,5	7

Test 1-6

Activa	Ketac
9	7,5
6	8,5
7	9
7,5	9

Test 1-7

Activa	Ketac
6,5	10
7	9
7,5	8
7,5	8,5

Test 1-8

Activa	Ketac
10,5	12
10	9
7	8,5
7,5	7

Test 1-9

Activa	Ketac
8	13
8	7,5
7	7,5
7	8

b) Test 2 – Second repetition

Test 2-1

Activa	Ketac
7	10
7	7
9	8,5
8,5	6

Test 2-2

Activa	Ketac
9	9
6	8,5
6,5	7
7	10

Test 2-3

Activa	Ketac
6	6,5
6,5	6,5
8,5	7
9	8

Test 2-4

Activa	Ketac
7,5	9
7	9
9	8,5
6	6

Test 2-5

Activa	Ketac
8,5	6,5
9	13
8	12
7	10,5

Test 2-6

Activa	Ketac
8,5	10
8	9
7	6
9	11

Test 2-7

Activa	Ketac
6	8
8	7,5
8	9
8	7,5

Test 2-8

Activa	Ketac
9	10
7,5	8
8	9
8,5	8

Test 2-9

Activa	Ketac
6,5	8
10	9
8,5	6,5
8	7

c) Test 3 – Third repetition

d) Test 4 – Fourth repetition

Test 4-1

Activa	Ketac
6,5	8,5
7	10
7,5	9,5
8	10,5

Test 4-2

Activa	Ketac
8,5	7,5
8,5	9
8	9,5
8	9

Test 4-3

Activa	Ketac
7,5	8
7	8,5
9,5	9,5
8,5	9

Test 4-4

Activa	Ketac
7,5	11,5
6,5	8,5
7	9
7	9

Test 4-5

Activa	Ketac
7	9
8	8,5
9	8
9,5	8,5

Test 4-6

Activa	Ketac
8	8
8	9
8,5	9,5
9	8,5

Test 4-7

Activa	Ketac
8,5	10,5
9	10,5
8	9,5
10	9,5

Test 4-8

Activa	Ketac
7,5	9,5
7	8,5
7,5	7,5
9	9

Test 4-9

Activa	Ketac
10	7
6	8
11	8
9,5	7

e) Test 5 – Fifth repetition

Test 5-1

Activa	Ketac
11	7,5
9,5	8
10	9
9	11

Test 5-2

Activa	Ketac
7	8,5
10	8
8,5	9,5
9	9,5

Test 5-3

Activa	Ketac
8,5	9
9	9
7	7
7,5	8,5

Test 5-4

Activa	Ketac
8,5	6,5
8	7
7,5	8,5
7,5	8

Test 5-5

Activa	Ketac
7	7,5
8,5	7,5
8	7
9	7,5

Test 5-6

Activa	Ketac
9	8,5
8,5	8
7,5	7,5
8	8

Test 5-7

Activa	Ketac
7,5	8,5
7	8
9	8,5
9,5	8

Test 5-8

Activa	Ketac
9	7,5
8	7
9,5	8,5
8,5	8

Test 5-9

Activa	Ketac
8,5	9
9	8,5
9,5	8,5
8,5	7,5

f) Test 6 – Sixth repetition

Test 6-1

Activa	Ketac
8	8,5
8,5	9,5
9	10
9,5	7,5

Test 6-2

Activa	Ketac
8	8,5
9,5	8,5
9	7,5
8,5	7,5

Test 6-3

Activa	Ketac
7,5	8,5
7,5	8
8,5	8
8,5	9

Test 6-4

Activa	Ketac
9	7
8	8
7	7
8	7,5

Test 6-5

Activa	Ketac
8,5	8
7	8
7,5	8,5
7,5	7

Test 6-6

Activa	Ketac
7,5	9
7	7,5
8,5	7,5
7	8,5

Test 6-7

Activa	Ketac
8	9
8,5	7
7	7
8	7

Test 6-8

Activa	Ketac
8	8
7,5	8
8,5	8
8	8,5

Test 6-9

Activa	Ketac
7,5	8,5
7	7
8	7
8	8,5

g) Test 7– Seventh repetition

Test 7-1

Activa	Ketac
7	8,5
6,5	8
8,5	8
9	8

Test 7-2

Activa	Ketac
8	9
8	8
8,5	7
8	8

Test 7-3

Activa	Ketac
8,5	8
8	8
8	7,5
8,5	7,5

Test 7-4

Activa	Ketac
8	9,5
7,5	8
7,5	9
8	9,5

Test 7-5

Activa	Ketac
8	8
8,5	8
9	8
9	8,5

Test 7-6

Activa	Ketac
7	11
7,5	12
7	8
8	10

Test 7-7

Activa	Ketac
8	9
9	8
9	9
9	8

Test 7-8

Activa	Ketac
9	10
8,5	10
8	9
8	9

Test 7-9

Activa	Ketac
9	8,5
9,5	9
8	9,5
8,5	9
